# The use of dynamic elastomeric fabric orthosis suits as an orthotic intervention in the management of children with neuropathic onset scoliosis: A retrospective audit of routine clinical case notes

**DOI:** 10.1186/s13013-016-0073-z

**Published:** 2016-05-31

**Authors:** Martin Matthews, Suzanne Blandford, Jonathan Marsden, Jennifer Freeman

**Affiliations:** DM Orthotics Ltd, Cardrew Way, Cardrew Industrial Estate, Redruth, Cornwall TR15 1SS UK; Faculty of Health and Human Sciences, Peninsula Allied Health Centre, Plymouth University, Derriford Road, Plymouth, PL6 8BH UK

**Keywords:** Dynamic elastomeric fabric orthoses, Children, Neuropathic onset scoliosis, Orthosis, Lycra® orthoses

## Abstract

**Background:**

To date the main treatment approach for neuropathic onset scoliosis has utilised thoracic lumbar sacral orthoses (TLSO) to stabilize the spine and enable stable sitting. Dynamic elastomeric fabric orthoses (DEFOs) may achieve both of these aims if used as an early intervention. Due to a lack of evidence in this area, a retrospective audit of case notes was undertaken to understand current orthotic practice investigating the usage, outcomes and clinical characteristics of treated children with neuropathic onset scoliosis. Clinical notes of 180 children at risk for, or identified with, scoliosis were audited using a search matrix to identify diagnostic group, spinal muscle tone, Gross Motor Functional Classification Scale (GMFCS) level, orthotic treatment modalities, scoliosis specific data, surgical interventions, adaptive technologies used, and outcome measurements reported.

**Results:**

Of the 180 notes examined, 85 were male; mean age nine years one month [SD four years seven months]. Spinal muscle tone was reported in 137 cases: 122/137 presented as low tone, 4/137 high tone, 6/137 fluctuating tone and 5/137 typical tone. Scoliosis was confirmed in (77/180) of whom (39/77) used a DEFO. Another (43/180) had a spinal curve developing, of whom (22/43) used a DEFO. The remaining (60/180) had no report of spinal curvature, but used a DEFO as a preventative measure. GMFCS scores were reported for 49 children of whom 14/49 were graded as level 4 and 17/49 level 5. Of the children with scoliosis who had spinal curve shapes reported, 48/60 had a C-shape presentation and 12/60 had an S-shape.

**Conclusions:**

The findings confirm previously reported papers in children with neuropathic onset scoliosis in relation to curve shape and GMFCS levels. It provides some evidence of the role DEFOs may have in the management of these children, and highlights the need for further research in this area due to the lack of peer-reviewed publications.

## Background

Neuropathic onset scoliosis, as classified by the Scoliosis Research Society (SRS), incorporates central and/or peripheral motor neuron involvement [1]. It can occur in children with cerebral palsy (CP), which can be attributed to non- progressive disturbances to the developing brain [2]. The condition affects two to three per 1000 live births and is recognised as a major cause of serious physical disability in childhood [3]. CP is often accompanied by impairments of sensation, cognition, perception, communication, and behaviour [2].

Neuropathic onset scoliosis occurs in boys and girls and can be attributable to disharmonious control of the trunk musculature around the spinal axis complicated by muscle compensatory mechanisms. Idiopathic scoliosis occurs predominantly in girls [4], however, incidence in the neuropathic onset group is unclear. It is understood in idiopathic scoliosis, that constant strong pathological pressure inhibits endochondral longitudinal growth on part of the spinal vertebra, whilst a contrasting reduction in compression results in accelerated eccentric growth, resulting in vertebral wedging [5]. The incidence of spinal deformity in children with cerebral palsy is reported as 25 %, ranging from 5 % for bilateral-spastic to 74 % in quadrilateral-spastic presentations [6]. Children who are wheelchair dependent due to a neuropathic or neuromuscular disease have a 90 % increased risk of progressive spine deformities due to impairments in postural balance and motor control [7]. Children with spastic CP have a 68 % chance of developing scoliosis, with Cobb angle progression of over 60° in 67 % of children with total body involvement; for those bed-ridden, the incidence can increase to 98° within three years [7, 8]. In contrast, ambulant children typically have only 9–15 % likelihood of developing scoliosis [9].

Severity of neuropathic onset scoliosis is linked to the Gross Motor Functional Classification Scale with evidence to suggest that children with level five classification will deteriorate at a faster rate than children with level one to four [10]. The curve presents as one of two variants; an S-shape with balanced, symmetrical thoracic kyphotic and lumbar lordotic curves [6] accounting for 20 % of spinal curvature, and a single thoracolumbar or lumbar C-shape associated with pelvic obliquity and hip dislocation [9]. The latter are experienced in the more severely affected wheelchair based patient (GMFCS level five), and are more likely to experience continuing Cobb angle increase [10]. Children with Retts Syndrome and scoliosis present with similar C- and S-shaped spinal curves as children with CP, and experience similar hip migration [11].

Children with CP presenting with GMFCS level four and five will at some stage require spinal surgery [12], and have lower functional results in spite of achieved fusion [7]. Often a child is placed in a wheelchair as the only option to provide stability and enable inclusion in activities of daily life. Seated children are more likely to develop curves secondary to the flexed sitting pattern that encourages spinal deformity based on atypical loading patterns and loss of the protective lordosis [13]. This is different from the more symmetrical physiological loading conditions in standing and walking where the spine is in extension. The asymmetrical seating position can also cause pain and discomfort, leading to an increasing decline in quality of life [13].

Stability of the spine relies on the interaction of both extrinsic (muscle force and gravitational effects) and intrinsic (vertebral structural interrelationship) factors [14] such as the counteracting growth forces seen when stretch growth of the nervous system is hindered [15]. The presentation of scoliosis is invariably shown as postural asymmetrical positioning of the spine due to secondary unbalanced muscle tone [4], coupled with the “vicious cycle” [5] of proprioceptive learning and spinal decompensation [14]. A child with low tone will tend to sit to one side or in kyphosis due to gravitational pull and lack of anti-gravity muscle activity. This sitting position is “learnt” by the child’s brain as correct sitting, resulting in asymmetry of pressure on the vertebral growth plates. The high pressure prevents growth of a portion of the vertebra, whilst the unloaded segment experiences accelerated growth; therein driving the “vicious cycle” [5], resulting in alteration to the brain’s self-image and perpetuation of the process [16]. These may result in extreme curve angles, coupled with excessive vertebral rotation, often in excess of 90°.

There is no convincing evidence in recent literature for the effectiveness of spinal orthotic intervention in the management of neuropathic scoliosis [17]. This, at least in part, is due to the complexity of the condition, and natural history of continual curve progression. In older literature, clinical experience considered the use of prolonged supine and prone positions, plaster shells or plastic orthoses after reposition under traction or on Risser traction tables to be effective. These practises have now been abandoned for reasons such as pressure sores and skin irritations [18]. It has been suggested that spinal orthoses may reduce the rate of curve progression [4, 6, 19, 20], and there is evidence to demonstrate that bracing can limit the consequences of impaired pulmonary development and function [1].

Rigid plastic spinal orthoses are custom-made orthoses that are manufactured from a plaster cast taken by the clinical orthotist, while the curve is corrected. The orthosis prescription is designed by the orthotist to counter the scoliosis presented, confirmed by X-ray blue-printing [21]. This type of bracing is the stalwart of idiopathic scoliosis management, however they are often too rigid for the neuropathic population as the patient is unable to move within the orthosis due to lack of strength and/or atypical tone. This may result in skin irritation or poor orthosis fitting that may be accompanied by heightened skin pressure areas over bony prominences, respiratory compromise, and feeding or swallowing disorders [22], all of which have the potential to reduce quality of life. As a consequence, poor compliance is common with orthosis use [6]. Despite product developments such as the use of soft, closed cell foam under arm spinal jackets to improve comfort [18], patients often experience similar issues to the rigid bracing because of the need to fixate the orthosis over the iliac crest to provide a basis on which spinal distraction occurs. Bracing is therefore mainly used to delay the inevitable surgical intervention and to improve wheelchair sitting ability [23].

Surgical intervention for scoliosis correction is common in this patient group, particularly the children in the GMFCS Level four and five classification [12] due to the speed of curve progression; therefore, regular spinal monitoring is required. Surgery often requires fixation of the spine via various instrumentation options that depend on scoliosis presentation and patient age. There is evidence to suggest that early surveillance of hip subluxation, coupled with early surgical intervention when required, can provide improved seating in the long term [24], although no link between scoliosis and hip asymmetry has been shown [10].

**Fig. 1:**
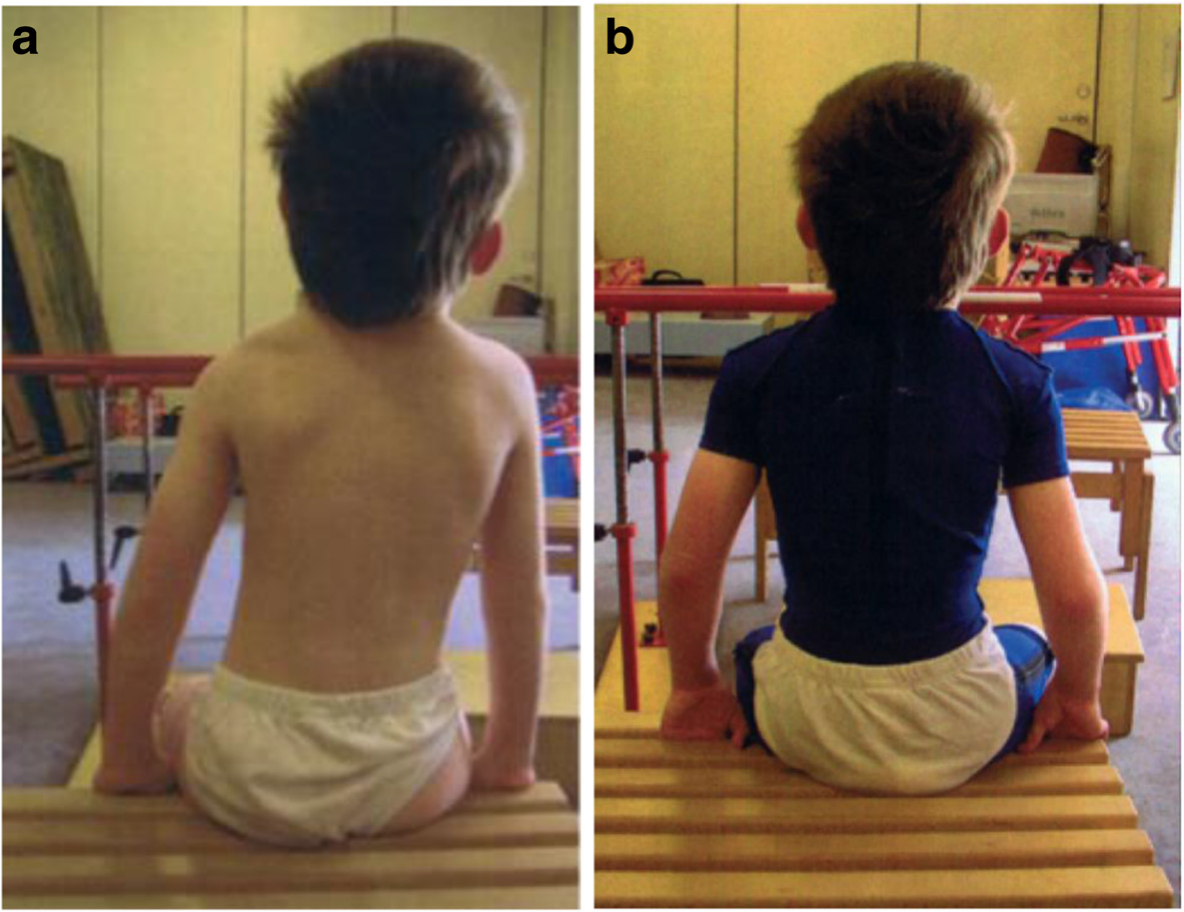
**a** Sitting position of child with cerebral palsy demonstrating inability of correction and awareness of body symmetry. **b** Sitting position of the same child wearing a customised Dynamic Elastomeric Fabric Orthosis suit

Dynamic elastomeric fabric orthoses (DEFOs, Fig. 1) have been used to stabilise the spine in children with CP for over 15 years. These custom-made orthoses, consist of a base layer of cotton based Lycra® fabric with strategically positioned reinforcement panels to provide specific areas of resistance to stretch, therefore the fabric possess reduced elasticity. These provide areas of high pressure, which are thought to increase proprioceptive input and produce a mechanical compressive effect. Detailed linear and circumferential measurements are taken by orthotists, who also specify the reinforcement panels required, based on clinical manuals and training programmes. Radiographic blue-prints are used to identify vertebral null points [21], along with Cobb angles, to prescribe the correct strength, direction and position of the reinforcement panels to counter the position (expert opinion of author MM).

Although clinical outcomes have been reported in various case studies [21, 25], there is a lack of published experimental evidence to enable robust evidence based practice. The use of DEFO suits is a continually progressive area of orthotic intervention for scoliosis. Therefore, the aim of this retrospective audit was to identify clinical data collected on the orthotic management of children with neuropathic scoliosis, to enable the description of clinical practice and outcomes across several centres. The purpose was to develop protocols for the use of DEFOs in children with neuropathic onset scoliosis.

## Method

### Data collection tool

A literature review, alongside discussion with specialist paediatric physiotherapists experienced in the use of DEFOs, provided a list of key data identifiers to examine clinical notes. This formed the basis of development of the interrogation tool with the aim of systematically collecting demographic (age, gender) and diagnostic characteristics (primary neuropathic diagnosis, co-morbidities, muscle tone, and GMFCS levels) allowing for a snap-shot of “care imparted”.

The following information was collected from physiotherapy, orthotic notes, and medical records for each year since birth:

1. Neuropathic characteristics: Gross Motor Function Classification Scale (GMFCS; Graded from Level 1 (the child can walk and climb stairs without limitation) through to Level 5 (indicating lack of independence even in basic anti-gravitational postural control)) [26], and muscle tone (low, high, fluctuating, and typical). It should be noted that recording of spinal muscle tone is difficult to categorise with any degree of accuracy due to the transient notation of clinical notes.
2. Functional status: head control, sitting, standing, and walking ability (recorded as unable, independent, independent with aid, or requiring assistance)
3. Scoliosis: scoliosis diagnosis confirmation, scoliosis level, curve presentation (“C” or “S”), Cobb angle in degrees taken from top and bottom vertebral end growth plates deemed to represent the single curve [27] (Scoliosis was accepted as Cobb angle ≥ 10 degrees.)
4. Prescribed mobility and seating systems
5. Additional use of health resources, including hospital admissions and reasons for admission, spinal surgical interventions, and use of hospital and/or community-based therapy services
6. Orthoses: type prescribed, change made, and problems reported

### Data collection

Case notes were obtained from five paediatric physiotherapy departments within National Health Service (NHS) hospital trusts across southern England who volunteered to be involved in the review. The therapists identified “any child with a neuropathic scoliosis of any severity, or perceived at risk of scoliosis development”. The primary source of data was from physiotherapy and orthotic notes (in either paper or electronic format) coupled with full medical notes when required, which covered a maximum of 10 years of clinical notes. Data was collected for each year from birth. Reliability of data collection was tested by comparing results of two researchers who separately extracted information from ten sets of notes; reliability was determined to be excellent with 98 % of all acquired fields being identically recorded. Thereafter, one researcher undertook data extraction. Any queries related to the notes due to legibility and such were discussed with the relevant therapy team on site.

This audit of clinical case notes was registered, and approved by the clinical audit departments for each of the participating hospital trusts. Funding was provided by the British Government under a Knowledge Transfer Partnership with Plymouth University (Project Reference: 100812) who carried out the study, which was undertaken independent of both the NHS organisations and DEFO manufacturer.

## Results

### Participants

The clinical note audit identified 211 suitable children, however 16 did not meet the criteria as they were either incomplete or illegible and 15 sets of notes were not found. The remaining 180 notes were analysed. Table 1 describes the demographic and diagnostic characteristics of the sample.

**Table 1:** Demographic and diagnostic characteristics of study sample (*n* = 180)

Age: mean years (+/- sd); *n* = 180	9 years (SD 4 years 7 months)
Gender (%)	53 % female
Diagnosis: *n* = 180	Cerebral Palsy: 79
	Neuromuscular Dystrophy: 5
	Developmental Delay: 42
	Others, e.g. Retts Syndrome, Epilepsy: 54 (30 %)
Gross Motor Function Classification Scale score: *n* = 49 (available score range 1 – 5)	Score 1: 2/49
	Score 2: 5/49
	Score 3: 11/49
	Score 4: 14/49
	Score 5: 17/49

### Spinal curvature and scoliosis

**Fig. 2:**
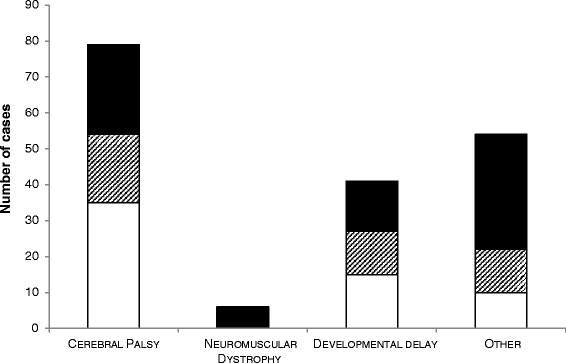
Primary diagnostic classification in 180 children: number without scoliosis (no fill), number with a spinal curve developing (*striped*) and number with scoliosis (*black*)

Figure 2 categorises the presence of scoliosis according to primary diagnostic classification.

Of the total sample (*n* = 180), 120 children were recorded to have either a confirmed scoliosis (*n* = 77) or curvature (*n* = 43) which was understood to be a kyphosis or low tone C- shaped curve. The average age of children with curvature or scoliosis development was seven years eight months (SD four years two months, *n* = 96/120) at first development of scoliosis. Scoliosis presentation was reported with C-shape 80 % (48/60) and S-shape (12/60). The curve level was identified in 52/77 (68 %) of the children: 23 (44 %) thoracic, 15 (29 %) thoracolumbar, and 14 (27 %) lumbar.

Seventy five percent of the notes of those with confirmed scoliosis (58/77) had sufficient data detail to identify the severity of the curves into low (5–24°), moderate (25–44°), severe (45–59°) and very severe (60° and above) [27]. Severity was identified, either by the largest Cobb angle reported over the years on X-rays or in the clinical notes (*n* = 42), or where this was not available, by radiologist reports describing the scoliosis as either mild, moderate, severe or very severe (*n* = 16). Based on this, 64 % (37/58) were recorded as mild, 28 % (16/58) moderate, 5 % (3/58) severe and 3 % (2/58) very severe scoliosis. The Cobb angle records (*n* = 42) indicated a mean maximal Cobb angle of 34.1° (SD 20.6°, range 9-100°). When categorized according to severity the mean Cobb angle was 17.3° for mild (*n* = 17/44, SD 4.9°); 31.0° for moderate (*n* = 16/44, SD 5.6 °); 50.2° for severe (*n* = 5/44, SD 4.1°) and 76.3° for very severe (*n* = 6/42, SD 16.0).

### Physical abilities

The GMFCS score was recorded in 49 of the 180 notes (refer to Table 1 for details). Scores were from across the available scale range; with two (4 %) children scoring the minimum level one and 17 (35 %) scoring the maximum level five.

Spinal muscle tone was recorded in the therapy notes of 137 children: 122/137 (89 %) were described as displaying low tone, 4/137 (3 %) high tone, 6/137 (4 %) fluctuating tone and 5/137 (4 %) typical tone.

### Scoliosis management using orthoses

**Fig. 3:**
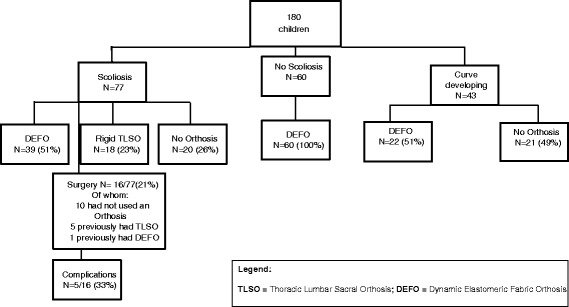
Schematic representation of the orthotic interventions used by children in the study sample (*n* = 180)

Figure 3 provides a schematic representation of orthotic interventions and surgeries used by the children within this sample.

#### Dynamic Elastomeric Fabric Orthoses (DEFOs)

Sixty children without scoliosis used DEFOs for preventative management, as did 22/43 with a developing curve. Of the 77 children with confirmed scoliosis, 39/77 wore DEFOs, although over time four children converted to rigid orthoses and four stopped wearing any orthotic intervention (one of whom was discharged as the scoliosis had disappeared). Of the other three, one child lost funding supporting the use of their DEFO, one-experienced fitting issues due to weight gain, and one was found to have no change in sitting posture with or without the suit.

By proportion, children with severe scoliosis were less likely to be prescribed DEFOs. Of those whose scoliosis was categorised according to severity, 34/42 with mild scoliosis used DEFOs, compared to 7/8 with moderate scoliosis and 1/8 with severe scoliosis. There was no record of using DEFOs in the very severe scoliosis category.

Details regarding when the DEFO was first prescribed were available for 38/39 children. The data from these children highlight that 26/38 had DEFOs provided following scoliosis diagnosis, while 12/38 used the DEFOs as a preventative measure. Of these 12 children, three had ceased wearing the DEFO before scoliosis diagnosis. There is no mention in the notes of parental compliance issues; however, this cannot be ignored as a possibility for non-compliance. For the remaining nine children, the average time of scoliosis diagnosis, post prescription was one year six months (SD 1 year 2 months, range 0.4-4 years).

Three out of the five centres had extensive experience over the previous 15 years of using DEFOs in the treatment of children with cerebral palsy. In the absence of national guidance in this area, locally developed written protocols were used to facilitate the identification of early prognosis of possible scoliosis due to low core (truncal) tone. These protocols are supported by unpublished local internal reviews on clinical outcomes and the reduction in the number of children with GMFCS level 4/5 who progressed to surgical intervention.

#### Rigid plastic spinal bracing

Rigid plastic spinal orthoses were used at some point in time by 18/77 children of whom 6/18 were still using them at the last datum point. Of the 12 children who ceased using a rigid orthosis, two converted to DEFO management, two did not comply with wearing the rigid orthosis, and the remaining eight were prescribed rigid orthosis management pre and post- surgery (three of whom were unable to tolerate the orthosis).

### Scoliosis management with surgical intervention

Surgery was performed in 16/77 of the children with scoliosis. The diagnoses included two CP, two neuro muscular disease, one Duchenne Muscular Dystrophy and six “other”. The mean age at surgery was nine years seven months (SD 4 years 5 months), the mean pre-operative Cobb angle was 62° (SD 26°, *n* = 8/15, range 72.5°). Prior to surgery 5/15 children had used a rigid orthosis, and up to three forms of postural management and one had previously used a DEFO. The remaining 10 children used no orthotic intervention. A further 7/77 children were awaiting spinal fixation surgery: two children had a diagnosis of CP, three NMD and two “other”; with a mean age of 13 years seven months (SD 2 years 5 months). Of the 16 children who underwent surgery, complications occurred in six and included rod breakage, excessive movement of fixations, infection, reduced head control, respiratory arrest, and deep wound infection.

### Progression of scoliosis in children with recorded Cobb angles

Cobb angles were reported for 42 children, with 18/42 having Cobb angles recorded at several time-points over a number of years. For these children the scoliosis angle increased at an average 6.5° (SD 9.7°) per year. A further 8/42 children had annual reviews. Scoliosis improved in 6/26 children and three of them no longer needed any intervention. These three children were managed by DEFO scoliosis suits, with a scoliosis progression of -6.3° (SD -6.7°) over an average of one year, eight months (SD 5 months). A further five children who wore DEFO suits maintained their scoliosis with no progression over an average of one year, four months (SD 11 months). The other 15/42 children had curve progression with the main management method being surgical intervention.

## Discussion

We are unaware of any other multi-centre review of clinical notes that gathers information on the use of DEFOs and orthotic management in the prevention and management of paediatric neuropathic scoliosis. The five centres in the south of England used differing preventative and scoliosis management interventions in this review, which highlights variation in practice between services. This “post-code lottery” of UK health service provision is commonly referred to in the literature [28].

The orthopaedic journals have discussed the outcomes of neuropathic scoliosis management using spinal correction orthoses [1, 29–31]. Several papers questioned whether dynamic orthoses worked in the long term [32, 33], whilst one author described them as “illogical in patients with neuromuscular scoliosis as these orthoses require normal muscle function to be effective” ( [20]; pg314). The term “dynamic” can be described as a force that initiates change, where the effect is likely to be constrained by the linear range of the elasticity of the fabric [34]. The fabrics possess dynamic elastic properties [34], which are measured as tension in Newtons [35]. Elastic fabrics are constructed of synthetic linear macromolecules of alternating hard and soft segments linked by urethane bonds [34]. The DEFO aims to achieve these properties by utilizing the basic compressive force provided by the base layer of fabric to adhere to the skin surface. Further layers of stretch resistive reinforcement Lycra ® applied to the base level fabric initiate a shear or compressive force through the skin to the underlying body structures, resulting in modified and improved spinal symmetry. The findings of this review of clinical case notes lends support to these positive outcomes.

While not directly associated to the findings of our study, we consider it important to outline aspects of our observational research and clinical knowledge, which we include in the paragraph to follow. Circumferential pressure has been shown to provide some stabilization to the spine [36] and to reduce pelvic pain [37] thereby providing an improved level of comfort whilst sitting. Improved trunk stability in the transverse plane [38] provides a stable basis to encourage spinal symmetry. A single case study demonstrated that improved stability can provide an opportunity to provide de-rotational coupling, with compression of the shoulders on high thoracic curves to enable a reduction in scoliosis [21, 22], providing the curve is mobile and not fixed due to bony deformity. Everyday comfortable sitting position is important to quality of life and to appropriate spinal growth and development. It has been suggested that an ideal position should be a slight lumbar lordosis with slight thoracic kyphosis [39]. Children with neuropathic onset scoliosis often present with a posterior sitting position [40], such that the centre of gravity is above or behind the ischial tuberosity with only approximately 25 % of the body weight transferred to the feet. If this is not corrected vertebral changes can occur due to asymmetrical loading to the vertebrae [5]. There is an increasing body of evidence that trunk postural control is an important determinant of motor function and that there is a precise relationship between control of the individual trunk segments and resultant effect on gross motor function and mobility [41]. It is possible that by enabling better alignment of positional indicators, DEFOs may improve co-ordination and subsequent function due to enhanced hip joint stability, force closure (compression of the pelvic compartment) and proprioception [37, 42].

We speculate that the use of scoliosis suits may have an effect on neuroplasticity enabling relearning of motor pathways based on repetitive posture change, via retraining of proprioceptive awareness [37, 43–45]; however, this has yet to be empirically proven. A number of children with CP, who are susceptible to scoliosis, present in the early stages of development with low tone which is particularly evident in their abdominal muscles, observable as flared ribs [46]. These children, who do not have adequate muscle strength to appropriately counteract gravity, and who typically present with muscle asymmetry due to the brain injury, adopt patterns such as atypical sitting postures and are unable to adjust their body position [43]. There is some evidence of changes in the central nervous system in children with CP, initiated by prolonged sitting in the sagittal contour whether in lordosis or kyphosis, causing compressional changes within the spinal cord. [47] The brain maintains and updates an internal model that is used to enable prediction of the required ideal muscle movements to achieve a motor end goal [48]. This internal model formulates a motor plan via the feed-forward motor command mechanism to achieve the goal. In the child with CP the potential deficits in motor planning may present as difficulty in anticipating hand grip or movement forces, a longer period of time taken to get to expected muscle target force and in the planning of sequential movement. This results in a poor internal model of the child’s musculoskeletal system, with the resultant lack of an effective motor plan for a particular motor goal [16, 49–51]. The child may attain the task, but in an atypical way. As children with Rett syndrome present with similar curves to children with CP they are currently assessed and classified in a similar way [11].

The lack of evidence examining spinal bracing suggests that there is little assessment of the sagittal profile and functionality of the spine in children with neuropathic onset scoliosis. The main accelerator of progression appears to be the constant slumped posture which cannot be counteracted in any orthosis, with exception to those that restore lordosis; these are inappropriate for small children [52, 53]. It is hypothesised that the provision of suits to stabilise the spine and prevent scoliosis initiation, may provide a mechanism both for preventing scoliosis progression and for enhancing the child’s ability to maintain a more typical trunk posture and isometric muscle contraction activity [54]. Our retrospective audit of clinical notes provides some indication that the DEFO suits may be effective in achieving this. Scoliosis suits may utilise therapeutic principles of proprioceptive enhancement, improving the spatial awareness provided by the compression to the spine [37, 55] coupled with downward compression on the shoulders and a firm fixation around the pelvis to provide postural stability. Postural stability, or balance is defined as the ability to maintain and/or regain the centre of mass within the base of support whether that is standing or sitting [56]. The authors suggest that, combined together, these elements provide a more stable and “safe” postural basis for the child with CP; further research is necessary to substantiate this.

In contrast, it is suggested that the rigid scaffolding provided by rigid/semi rigid spinal jackets in this patient group may reduce the need for muscle activation, with the potential that relative enforced immobility may further atrophy trunk muscles in children with CP, who are already weak due to their condition. This has been demonstrated in children wearing ankle foot orthoses in the long term [57]. Underlying asymmetrical spinal weakness may initiate a vicious cycle [5], continuing even when wearing the orthosis. Over time, the postural curve may continue to progress and lead to vertebral wedging, rib deformation and excessive spinal rotational components [5]. Although the orthosis is usually routinely re-cast based on X-rays to inform pressure positions, the orthosis at best slows the progression. This may be because the orthosis is treating the resultant Cobb angle and rotation, and not the primary cause, which may be muscle imbalance. We suggest that the DEFO scoliosis suit provides a midpoint between the compressive effect of deep compression of the basic suit (a suit without any counter rotation or lateral translation panels) and that of a rigid plastic spinal orthosis. By coupling the corrective reinforcement panel forces with the compression of the body segments to provide stability (the accepted three point pressure systems of high/low pressure variants) [21], the scoliosis suit appears to provide an improved effect on trunk symmetry. Of the children who did not have a confirmed diagnosis of scoliosis (either no curve or a developing curve), 82/103 had been prescribed DEFOs.

This clinical notes audit suggests that DEFOs have a place in the prevention and management of paediatric neuropathic scoliosis, perhaps offering an alternative to rigid bracing in children with mild/moderate neuropathic scoliosis. The vast majority of the children prescribed DEFOs were compliant with wear where only three of 121 children stopped wearing the DEFO over the course of the audit timeframe. With the exception of one child (who only used postural equipment), children whose scoliosis either improved or was maintained were all managed with a DEFO. In all of these cases, scoliosis was classified as mild or moderate.

Two individuals were identified in our audit and provide anecdotal evidence of the positive outcomes that can occur with DEFO use. One child with Crouzon syndrome presented at baseline with a 42° curve and was prescribed a DEFO, which maintained the curve for a year (45°; angles determined via radiography). However, after growing out of the suit (not replaced) the curve progressed to 55°. Thereafter, the child was prescribed a new DEFO suit and over the course of the following year, the angle was subsequently reduced to 46°. Another child diagnosed with developmental delay had a 30° Cobb angle at baseline and was prescribed a DEFO; one year later the curve had reduced to 15°, and 2 years on (after consistent wear) their scoliosis had further reduced to 8°.

In accordance with previous literature [1] our results highlight some of the problems associated with surgical intervention with 5/15 surgically treated children experiencing complications. A recent report estimated the cost of one spinal surgical intervention for correction of neuromuscular scoliosis as US$50,096 (equivalent to £32,000) [± $23,988 (£16,000)] inclusive of the implants, specialist nursing and recovery [58]. This high cost reinforces the need to reduce the number of surgical procedures undertaken by providing early intervention to reduce, and if possible prevent, scoliosis of neuropathic origin. The recent Braist report provided data to confirm that long term compliance in idiopathic scoliosis orthosis wear shows clear evidence of reductions of curve progression [59], as long as compliance is good. It is likely that this is also the case in neuropathic onset scoliosis.

The average age of scoliosis or spinal curve development was seven years eight months, which confirms the need for early intervention. The average rate of progression of all children with scoliosis was 6.2° per year regardless of the management approach, neuropathic condition, or age. This is higher than the previously reported average rates for CP [8, 30] of 4.2-4.5° per year. The progression rate for children with muscular dystrophy was high at 5.7° per year, increasing to 29° in children requiring surgery [60]. In this retrospective case note audit, children wearing DEFOs who were monitored regularly over time, only 1/8 experienced a deterioration of >10°. This finding potentially supports the role that DEFOs may have in scoliosis management. We acknowledge the small samples used in our study, and are therefore cautious in our interpretation.

The combination of cost and curve progression indicates that more should be done to promote prophylactic interventions in the early years, be it the use of DEFOs and/or early hip repositioning surgery to optimise sitting posture. Recently, proposals have been made that could provide a treatment template for routine hip surgery [61] coupled with improved sitting provision, to improve the quality of life for this group of children. Early conservative intervention such as the use of DEFOs, if proven effective, could be used as a prophylactic option as an alternative to rigid orthoses in some children, as a way to minimise the need for surgical procedures, which in themselves provide an increased risk of complication.

Our retrospective audit of clinical case notes was designed to obtain as much data as possible while optimising sample inclusion. Having access to a time span of 10 years and data collection from five NHS trusts enabled a sample of 180 data sets. The University researcher, employed to undertake an independent review of the notes, had been trained for the role prior to visiting each clinic. However, interpretation of the results was reliant on the data available in the case notes, which was variable. While this might be considered a typical limitation of retrospective audits of healthcare records, consistency in completing case notes should be promoted across all contributing professions to improve comparative ability. There is a discussion to be had in relation to possible templates for therapy note keeping in relation to scoliosis management to ensure the basic data of Cobb angle, Risser sign (if appropriate) and regular follow up periods with X-rays is recorded, in addition to routine recordings of GMFCS levels. Nevertheless, our systematic approach enabled us to provide a snap-shot of the current management of children with neuropathic onset scoliosis in five NHS trusts in the south of England.

Future research, using methodology such as a prospective longitudinal design, should investigate the prophylactic effect of the DEFO in the development of neuropathic onset scoliosis. This future research should also further explore the mechanisms underpinning the effect of the DEFO intervention. Comparison of the DEFO and other scoliosis support systems and orthoses should also be investigated including detailed cost benefit analyses.

## Conclusions

This retrospective audit of current practice indicates that DEFOs are used in the management of mild to moderate scoliosis in a range of neuromuscular conditions, predominantly in CP. It has highlighted the large variation across different NHS services in the south of England and the potential need to work towards developing a national strategy for children with scoliosis. Cobb angle progression (where serial monitoring was performed) was typically minimal in those children managed by the DEFO. This provides some evidence for the potential role that DEFOs may have in the future management of children, both at risk and presenting with neuropathic onset scoliosis. Further research is required to establish effectiveness as proposed above.

## Consent

Written informed consent was obtained from the patients and their legal guardian for publication of this case audit and any accompanying images. A copy of the written consent is available for review by the Editors-in-chief of this journal.

## Abbreviations

CP: cerebral palsy
DD: developmental delay
DEFO: dynamic elastomeric fabric orthosis
GMFCS: Gross Motor Function Classification Scale
GMFM: Gross Motor Function Measure
NMD: Neuromuscular Dystrophy
SD: standard deviation (shown as SD 4 years 7 months)
SRS: Scoliosis Research Society
TLSO: thoracic lumbar sacral orthosis
